# Application of Internet-based multidisciplinary management in patients with genitourinary cancers in China: A brief introduction to a new model of healthcare service for cancer survivors during COVID-19 pandemic

**DOI:** 10.3389/fpubh.2022.952739

**Published:** 2022-10-06

**Authors:** Yu Zhu, Shengming Jin, Hangcheng Fu, Hailiang Zhang, Xiaolin Lu, Chengyuan Gu, Weijie Gu, Fangning Wan, Weiyi Yang, Xiaojian Qin, Dingwei Ye

**Affiliations:** ^1^Department of Urology, Fudan University Shanghai Cancer Center, Shanghai, China; ^2^Department of Oncology, Shanghai Medical College, Fudan University, Shanghai, China; ^3^Fudan University Prostate Cancer Institution, Shanghai, China; ^4^Shanghai Genitourinary Cancer Institution, Shanghai, China; ^5^Department of Urology, University of Louisville, Louisville, KY, United States

**Keywords:** Internet-based multidisciplinary management, COVID-19 pandemic, cancer survivors, genitourinary cancers, a new model of healthcare service

## Abstract

The coronavirus disease 2019 (COVID-19) pandemic has triggered multiple global healthcare system crises. Apart from the pandemic itself, the travel restriction and social distance policy for the purpose of epidemic control has cast a shadow on the management of cancer survivors. Cancer survivors suffered a double blow from both the epidemic and cancer. To deal with the challenge, we explored a new Internet-based patient management model. This model has overcome the limitation of time and space and thus can help oncologists to provide remote multidisciplinary healthcare services for cancer survivors. These patients can get high-quality cancer management from multidisciplinary experts without too much transportation. This model has been applied in patients with genitourinary cancers and proved to be effective and efficient. Our study demonstrated that more patients benefited from this model during the pandemic of COVID-19, especially in those affected heavily by COVID-19. These results suggested that it can also give insight into the management of other cancer survivors in China. Given the long-term impact of the COVID-19 pandemic, we would like to introduce our new model of healthcare service and the application of Internet-based multidisciplinary management to our global peers and medical industries to help their cancer survivors who are delayed in treatment due to the COVID-19 pandemic.

## Introduction

The coronavirus disease 2019 (COVID-19) pandemic has caused multiple healthcare system crises all over the world (1). The number of severe, critical, and dead patients caused by the pandemic varies in different regions, but all these cases caused a great burden on the corresponding regional and global medical services (2). This not only affects the control of COVID-19 but also squeezes the resources for diagnosis and treatment for other diseases, such as cancer. The restriction on the flow of population for the purpose of epidemic control makes it even more difficult to manage cancer patients, who suffered from both the epidemic and cancer (3, 4). After being hit by COVID-19 in China, we carried out strict policies to control the epidemic quickly and effectively and gradually restored normal daily life from the epidemic (5). China experienced the same pain as the vast majority of countries and regions all over the world, and Chinese cancer survivors and medical practitioners have also experienced a similar difficult situation that the world is experiencing (6, 7).

To cope with this situation, we explored a new Internet-based patient management model. In our study, we compared the overall situation of it before and after the outbreak of COVID-19. We also explored the operation status of our remote multidisciplinary team (MDT) platform in different provincial regions in the first 3 months of the COVID-19 pandemic. It demonstrated that this model has overcome the limitation of time and space and thus can help oncologists to provide remote multidisciplinary healthcare services for cancer survivors. These patients can get high-quality cancer management from multidisciplinary experts without too much transportation. This model has been applied in patients with genitourinary (GU) cancer and has proved to be effective and efficient. It can also give insight into the management of other cancer survivors in China. Given the long-term impact of the COVID-19 pandemic, we would like to introduce our new model of healthcare service and the application of Internet-based multidisciplinary management to our global peers and medical industries to help their cancer survivors who are delayed in treatment due to the COVID-19 pandemic.

## Materials and methods

We developed an online platform in April 2017 for MDT management for GU cancer across China called “China Genitourinary Cancer MDT Platform (CGCMP).” A typical implementation could be demonstrated as follows: Each medical center with GU MDT builds a local MDT expert database in advance. A chief expert is elected in each center to represent and lead the team in a remote MDT consultation between two centers. All involved MDT experts are certified from CGCMP. The chief expert will recruit experts' case sensitively. Patient information is uploaded from a local center. A secretary, usually a urology resident, is assigned by the local chief expert and will coordinate the MDT consultation, such as essential medical record preparation, new cases submission, case presentation, discussion recording, and communication with patients. A scheduled online consultation meeting for multiple cases is carried out with the support of a remote video conference system. When the chief experts from both centers reach a conclusion at the end of discussion, a summary of the diagnosis and management strategies will be generated for each case. The local secretary will explain the experts' opinions and deliver the summary to each patient. Our platform also supports online meeting models for multiple center discussion, inter-center discussion, individual-to-individual discussion, and discussion between individual experts and their teams.

**Figure 1:**
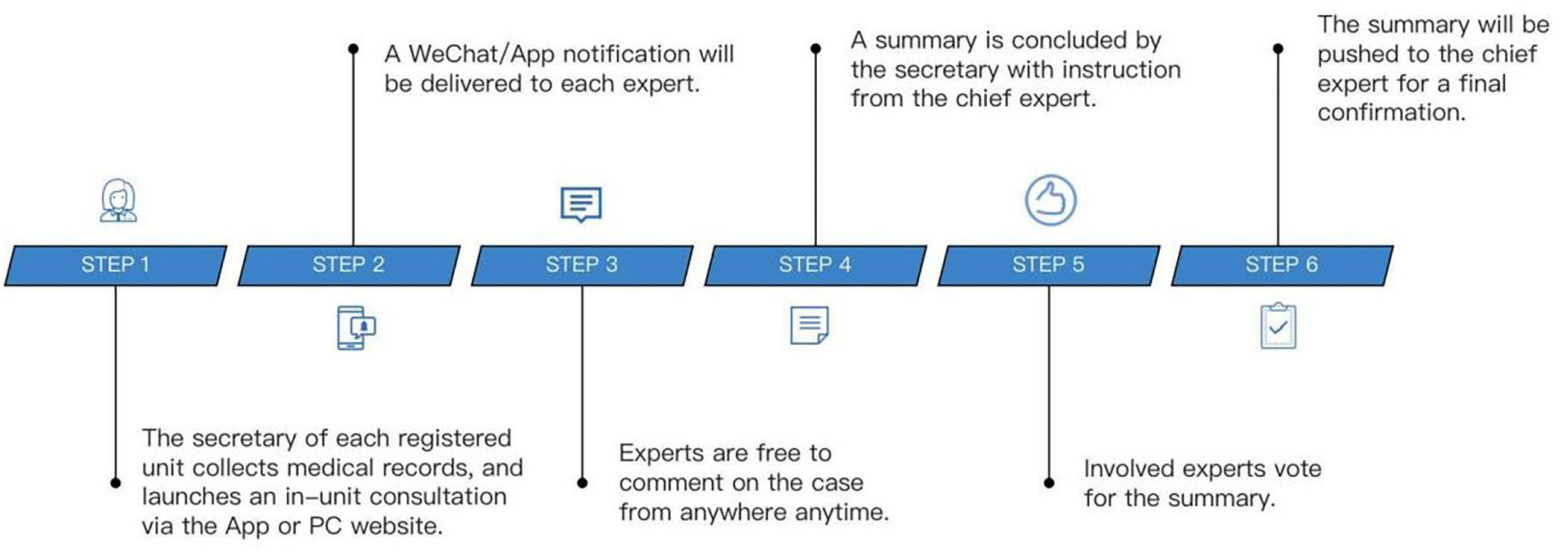
Work flow of the multidisciplinary team (MDT) system in a single center.

Further, we developed a mobile version application running on WeChat App, which is the most widely used social media service in China. Some well-known medical centers with great prestige in GU MDT services are titled regional centers, while other medical institutions that routinely provide MDT services are titled city centers. The secretary of each center is designated to collect medical records and submit an in-center consultation application *via* the Mobile App or PC Website. The Mobile App will forward a WeChat notification to each expert in the team once a new application is submitted. Experts are free to comment on the case from anywhere and anytime. The secretary will draft a summary, and then, the chief expert will review it. A final confirmation will be added by the chief upon agreement among all the experts. The workflow of the MDT system in a single center was illustrated in Figure 1. On the contrary, inter-center consultation is also encouraged and processed on the platform as stated above and coordinated by the platform.

Before the outbreak of COVID-19, the CGCMP system has successfully carried out more than 230 local and remote MDT consultations; more than 4,300 GU experts from 185 medical centers with GU MDT services in China are registered in the system. A preliminary hierarchical medical network, consisting of 4 regional centers and 118 city centers, is established, covering 30 of 34 provincial-level administrative regions in China.

Based on our previous work, a public welfare project to help our patients, called *China GU MDT Charity Campaign*, was initiated and launched by the Chinese Anti-Cancer Association-Genitourinary Oncology Committee (CACA-GU), Chinese Society of Clinical Oncology Prostate Cancer Committee (CSCO-PC), Urologic Chinese Oncology Group (UCOG), and Shanghai Genitourinary Cancer Institute (SHGCI) on 10 February 2020.

We made adjustments to the system to facilitate our patients to submit cases and get feedback effectively: The medical record uploading process was designed into element-based blocks, and layman's terms were used to help the patients to understand which attachment to upload and what text to enter in each block. For the text description parts, we categorized them into the pathological report, diagnosis, CT report, and reason for consultation, etc. We also set a minimum and a maximum word limit to ensure the conciseness of the uploaded record. Moreover, a remark text box was added for these non-medical professionals to express those personalized needs in their language.

A core operation team of 11 volunteers was set up, such as quality control expert, volunteer coordinator, consultation secretary, recorder, and technical support. Other volunteers who were participating in the charity campaign were recruited, and they were all well-known experts in the field of MDT services of GU cancer in China. CACA-GU, CSCO-PC, UCOG, and SHGCI also established an expert committee for the campaign to ensure the professionalism of consultation services. For each online MDT meeting, we established a temporary coordination team through the WeChat application, such as the 11 core operation volunteers and other volunteering experts and their assistants.

The first stage of the charity campaign ended on 27 February 2020. Due to traffic restrictions, experts in GU cancer in China took advantage of resources and technologies for multisite, multidisciplinary collaboration in remote medicine and used the platform with mobile devices during their break time to provide free consultations for patients with GU cancer across the country. After each consultation, the platform guided the patients to their nearest hospital in our system for further treatment or follow-up.

With the alleviation of the epidemic, transportation and medical resources are gradually released; we are able to meet the demand of more patients and also the need of continuing education for medical staffs. In the second stage of the charity campaign, experts in urological oncology across China continued to explore various models of charity campaign to better serve the patients and medical staffs by using Internet-assisted multidisciplinary services. In another dimension, we fully supported the frontier of anti-COVID-19 pandemic, provided a strong backup for patients with GU cancers, and also conveyed the latest concepts and experience to GU practitioners nationwide.

Some of these charity activities were carried out for the entire GU cancer survivors as a group. Through typical case presentation and discussion after desensitization of personal information, pre-release advertisements, live video broadcasts, and replays, we published anti-epidemic and medical guidance information to all concerned families in time to help them seek anti-cancer treatment while strengthening anti-epidemic measures and guided them to seek safe and effective medical treatment. Some were personalized, that is, for some complicated cases, patients would be invited to join the video consultation process to directly communicate with volunteers of MDT experts in an online meeting. MDT experts gave diagnosis and treatment advice and informed patients of the nearest medical institution, which could implement consultation advice.

**Figure 2:**
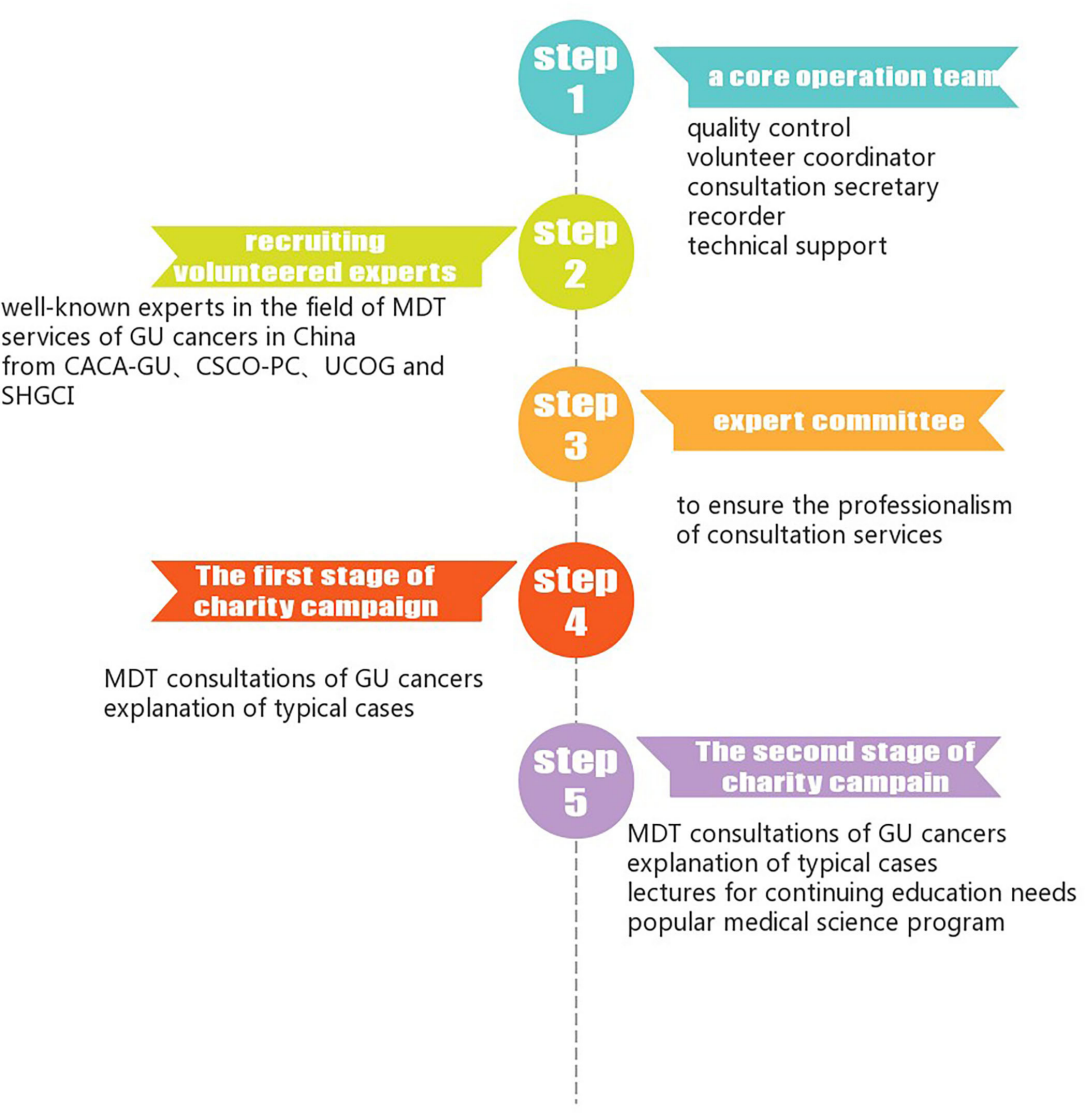
Work flow of China genitourinary (GU) MDT charity campaign.

In the second stage of the charity campaign since 28 February 2020, some activities were aimed at medical staff with continuing education needs. The volunteers of MDT experts gave lectures on the latest diagnosis and treatment in the field of GU cancers to the registered medical staffs through the platform. These cloud video meetings had different medical topics and also provided pre-release advertisements, live video broadcasts, and replays for registered medical staff. The workflow of the charity campaign is illustrated in Figure 2.

Our charity campaign also provided popular medical science programs. To ensure that more people could benefit from our charity activities, we chose different types of cooperative media on different topics, such as professional media, social media, health-related public media, or general public media.

We employed the ZOOM App as our core online conferencing system. The case collection system was developed by ourselves. During the case collection process, the patients were fully informed and well-consented that the information provided was only used for the charity campaign and would be properly kept. The sensitive information related to patient privacy was not collected.

## Results

### Overall situation of the Internet-based multidisciplinary management in patients with genitourinary cancer in China

In the first stage of the campaign, with the main purpose of solving urgent medical problems, we provided remote MDT consultation services to 391 patients with difficult cases and their families and assisted them to find the nearest medical center to carry out MDT consultation conclusions and receive corresponding diagnosis and treatment.

Among the 391 cases, 348 were men, with a median age of 65 years, and 43 were women, with a median age of 56 years; a total of 220 cases of prostate cancer, 94 cases of kidney cancer, 65 cases of bladder cancer, five cases of testicular cancer, two cases of penile cancer, and five cases of other cancers were discussed. The volunteer experts and their teams carried out 304 remote consultations through the smart system and fed back in time; by live broadcast, 87 typical cases, which were carefully desensitized, underwent MDT consultation by groups of GU experts, with more than 140,000 audiences. The patients who provided cases for consultation came from 28 out of 34 provincial administrative regions in China.

As of 30 April 2020, there are 334 GU MDT experts participating in 72 charity activities through the platform. Teams of experts discussed 659 difficult cases of GU cancer and arranged follow-up treatment nearby. At the same time, 87 online keynote lectures in the area of GU cancer were provided to professional audiences with continuing education need and received 495,534 visitors. Two short videos on public science education were released, combined with patient education activities, to more than 3.5 million non-professional visitors.

### Comparison of the operation status of the Internet-based multidisciplinary management in patients with genitourinary cancer in China before and after the outbreak of COVID-19

**Figure 3:**
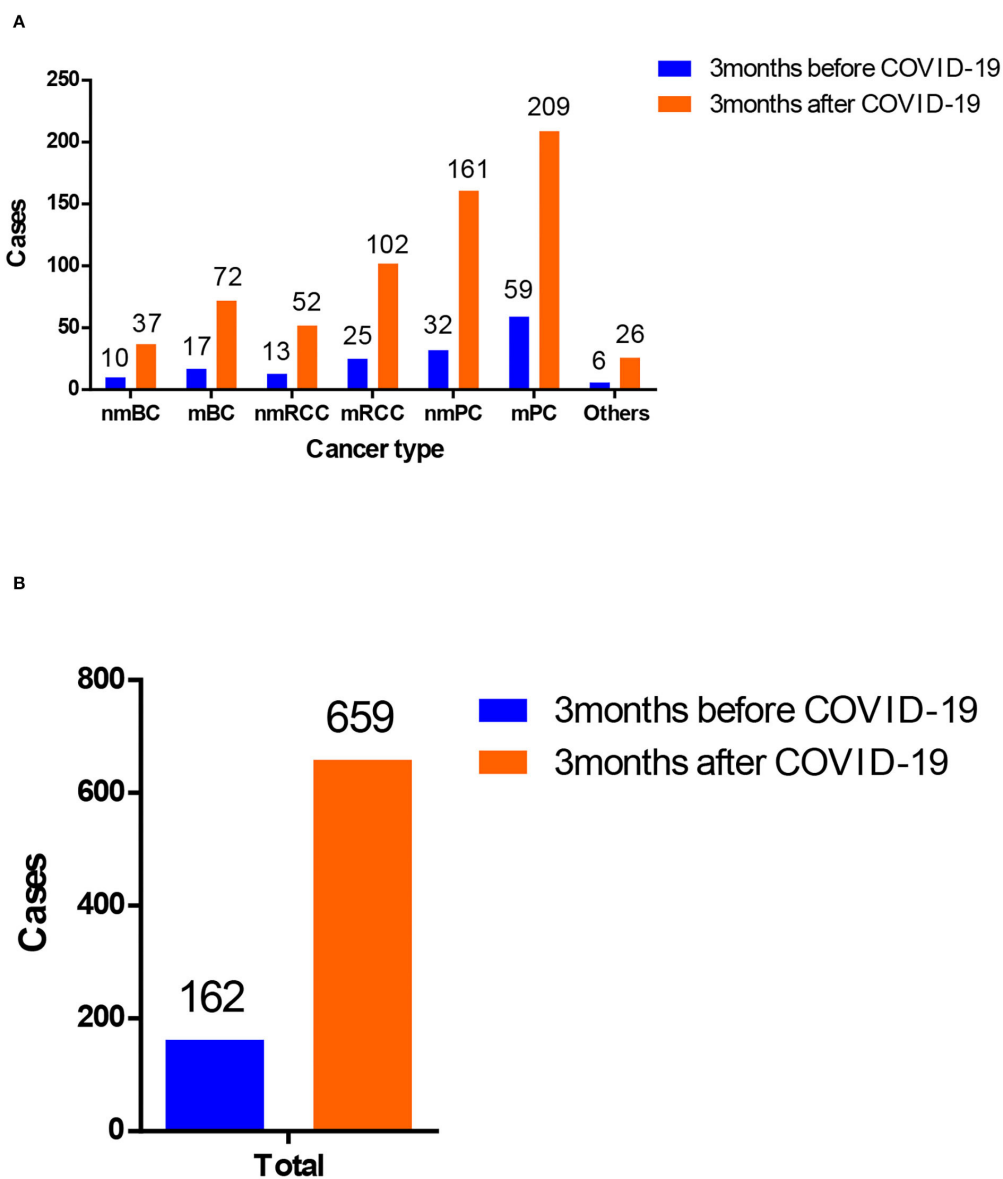
Comparison of patients with genitourinary cancer receiving medical consultation from online MDT platform in China before and after the outbreak of coronavirus disease 2019 (COVID-19). **(A)** Patients with different cancer types of genitourinary cancer and **(B)** overview of patients with genitourinary cancer.

To better characterize the benefit of our remote MDT consultation service, we compared the overall situation of it before and after the outbreak of COVID-19 (Table 1 and Figure 3). The total number of cases receiving online MDT consultations increased a lot after the pandemic. In 3 months before COVID-19, 162 cases received remote MDT consultation services. In addition, the number increased to 659 in 3 months after COVID-19.

**Table 1:** Comparison of the operation status of the Internet-based multidisciplinary management in patients with genitourinary cancer in China before and after the COVID-19 outbreak.

	**3 months before COVID-19 (Oct–Dec)**	**3 months after COVID-19 (Feb–Apr)**
**Situation of online MDT platform**		
Total number of online MDT activities	13	72
Number of professional visitors	20,192	105,739
Number of non-professional visitors	176,027	974,920
Total number of visitors	200,198	1,080,659
Number of participating medical centers	33	105
**Evaluation of effects from online MDT platform**		
Ratio of affirmative conclusions^a^	124/162	598/659
Ratio of improvement in diagnosis^b^	119/162	577/659
Ratio of improvement in management strategy^c^	113/162	586/659

a It means that uncertain conclusion was turned into affirmative conclusions after the MDT consultation.

b It means that the diagnosis was changed or an ambiguous diagnosis was turned into a definite diagnosis after the MDT consultation.

c It means that management strategy was changed or an ambiguous management strategy was turned into a definite management strategy after the MDT consultation.

In terms of the situation of the online MDT platform, 13 online MDT activities were performed 3 months before COVID-19 in total. A total of 200,198 visitors watched the online MDTs activities, of whom 176,027 visitors were non-professional visitors. In contrast, 72 online MDTs activities were carried out 3 months after COVID-19. A total of 1,080,659 visitors watched the online MDTs activities. Among them, 974,920 visitors were non-professional visitors.

To evaluate the benefit of the remote MDT consultation service, we investigated the feedback from the participating patients. In 3 months before COVID-19, 124 of 162 cases received affirmative conclusions after the remote MDT consultation service. Diagnosis of 119 in 162 cases was improved, and the management of 113 in 162 cases was improved. In comparison, 598 of 659 cases received affirmative conclusions after the remote MDT consultation service 3 months after COVID-19.

We also distributed questionnaires to 33 medical centers participating in online MDT before the pandemic. A total of 29 medical centers performed offline MDT before COVID-19, while two more started to perform offline MDT after COVID-19. During the pandemic of COVID-19, more medical centers benefited from the remote MDT consultation service, such as understanding standard cases (from 27/33 to 32/33) and the process of MDT (from 28/33 to 33/33), improvement of clinical decision making (from 30/33 to 32/33), and patient satisfaction (from 28/33 to 31/33).

### Education to medical centers and professional staff in China before and after the outbreak of COVID-19

**Figure 4:**
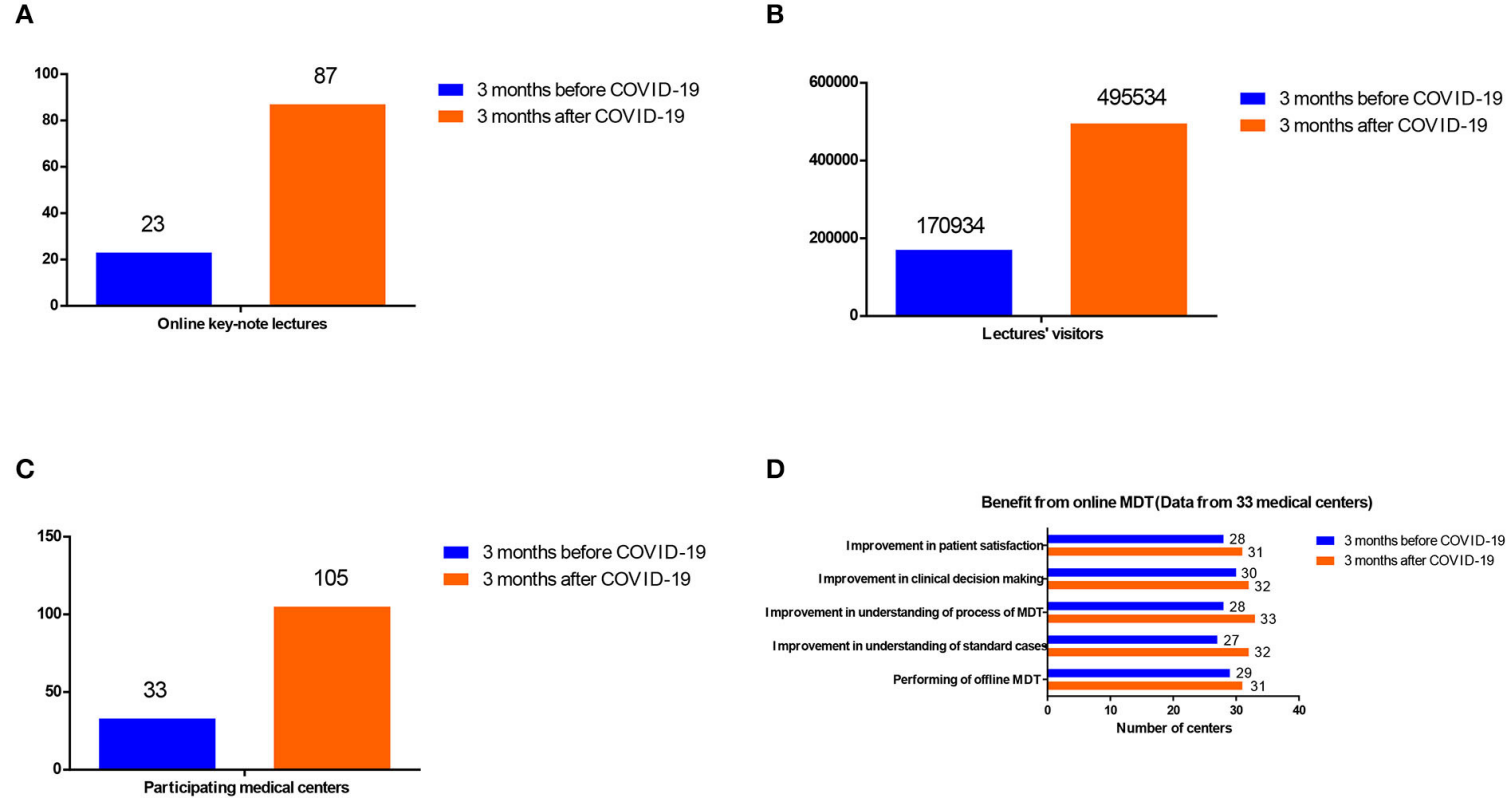
Comparison of the education role of the online MDT platform in China before and after the outbreak of COVID-19 **(A)** the online key-note lectures, **(B)** the lecture visitors, **(C)** the participating medical centers, and **(D)** by the benefit from online MDT (Data from 33 medical centers).

Besides the direct medical aid to patients, many medical centers and professional staff from different areas in China also benefited from our online MDT platform (Figure 4). In the 3 months before COVID-19, 23 online key-note lectures in the area of GU cancer were provided to professional audiences with continuing education need and received 170,934 visitors. In addition, 3 months after COVID-19, the number of online key-note lectures and visitors increased to 87 and 495,534, respectively. Also, the number of participating medical centers has also increased from 33 to 105. Thus, during the pandemic, our online MDT platform has played a greater role in educating professional audiences.

### Comparison of the operation status of the Internet-based multidisciplinary management in patients with genitourinary cancers in China in different provincial regions

To further investigate the role of our remote MDT consultation service has played during the pandemic, we compared the operation status of our remote MDT platform in different provincial regions 3 months after COVID-19 (Table 2). Provinces in China were categorized into two types: Hubei Province and other areas. It demonstrated that nearly 17% (110/659) of cases were from Hubei Province during the pandemic. Approximately 16.7% (180,209/1,080,659) of online visitors were also from Hubei Province.

**Table 2:** An overview of the operation status of the Internet-based multidisciplinary management in patients with genitourinary cancer in China by different areas during the COVID-19 epidemic.

	**Hubei province^**d**^**	**Other areas**
**Situation of cases receiving online MDT consultation**		
Total numbers of cases	110	549
Number of non-metastatic bladder cancer cases	7	30
Number of metastatic bladder cancer cases	14	58
Number of non-metastatic kidney cancer cases	10	42
Number of metastatic kidney cancer cases	18	84
Number of non-metastatic prostate cancer cases	27	134
Number of metastatic prostate cancer cases	35	174
Number of other cancer cases	4	22
**Situation of online MDT platform**		
Total number of online MDT activities	13	59
Number of professional visitors	17,630	88,109
Number of non-professional visitors	162,501	812,419
Total Number of visitors	180,209	900,450
Number of participating medical centers	16	89
**Evaluation of effects from online MDT platform**		
Ratio of affirmative conclusions^a^	99/110	499/549
Ratio of improvement in diagnosis^b^	106/110	471/549
Ratio of improvement in management strategy^c^	101/110	485/549
**Results based on questionnaires from participating medical centers (rated by 1–5)**		
Difficulty in performing offline MDT during the epidemic (1 is unchanged, 5 is very difficult)	4.9	3.8
Difficulty in performing online MDT during the epidemic (1 is unchanged, 5 is very difficult)	3.2	2.2
Difficulty in implementing follow-up decisions from MDT during the epidemic (1 is unchanged, 5 is very difficult)	4.8	4.2
Difficulty in implementing drug treatment decisions from MDT during the epidemic (1 is unchanged, 5 is very difficult)	4.9	4.3
Difficulty in implementing surgical and referral decisions from MDT during the epidemic (1 is unchanged, 5 is very difficult)	4.8	4.4

a It means that uncertain conclusion was turned into affirmative conclusions after the MDT consultation.

b It means that diagnosis was changed or an ambiguous diagnosis was turned into a definite diagnosis after the MDT consultation.

c It means that management strategy was changed or an ambiguous management strategy was turned into definite management strategy after the MDT consultation.

d The most affected province in China during the first 3 months of the pandemic.

Questionnaires were also distributed to evaluate the difficulty in performing MDT during the epidemic. It was very difficult to perform offline MDT in Hubei Province in the first 3 months of the pandemic. Furthermore, it was also hard to follow the advice from the online MDT during the epidemic, such as follow-up, drug treatment, surgical, and referral decisions in Hubei Province. This was confirmed when comparing results from other areas.

## Discussion

During the outbreak of the COVID-19 and the epidemic control policy, Internet-based medical services played an important role in the management of health (8). Compared to other online medical service platforms, our online consultation system combined with local medical services, although not designed for the pandemic situation, functioned well in solving some urgent medical needs in this special period of time.

We are not just an online platform providing consultation service but also by a platform enhanced by high-quality MDT management in the field of GU, which was implemented offline locally in the nearest medical center, authorized, and registered in our platform. The management of cancer required multidisciplinary intervention to obtain the best therapeutic effect (9). GU cancer has different biological behavior and different sensitivity to systemic treatments (10). Part of GU cancer survivors can obtain long-term control even in the late stage, while some others can only obtain palliative effects (11). With the improvement of medical technology and the innovation of tumor prevention and control concepts, more and more patients with GU cancer can achieve long-term survival, and the management of cancers has changed from a single dimension to a whole process (12).

In recent years, with the promotion and application of the MDT management model, the therapeutic effect of GU cancer has been significantly improved, and the need of the general population for health management has been initially met (13). However, due to the regional imbalance of domestic medical resources and medical staff training, the development of high-level, homogenized, and wide-ranging GU MDT services across the country still faces challenges in all aspects, such as technology, model, system, and policy (14).

To cope with the needs of medical treatment in this special period of time, our charity campaign made some improvements to the traditional model. Our charity campaign is based on a mature multidisciplinary platform for GU cancer. On this platform, we have the best GU MDT experts and their teams across the country and can achieve cross-regional cooperation through platform services. Relying on this platform and the consultation system, we can achieve consistent high-level diagnosis and treatment service in all regions of our country and also achieve localized medical services by regional centers and urban centers across the country.

Under the epidemic, technology has given new channels for medical healthcare. The resources and technical advantages of the remote medicine and multidisciplinary collaboration platform are fully utilized (15). CGCMP owes the following technical advantages: unified data management, flexible models for discussion, reliable remote video conference system, efficient coordination, and convenient participation.

The positions of the internet medical model in the future medical system should be recognized. In the last few years, technological developments in the medical field have been rapid and are continuously evolving. One of the most revolutionizing breakthroughs was the introduction of the Internet of things (IoT) concept within the medical practice (16). Due to its availability, IoT has played a more and more important role in the application of Internet-based multidisciplinary management in patients with cancers. Nowadays in many cancer centers in China, patients with cancers in remote areas can receive online consultation from experienced doctors, which promotes the availability of high-level management of cancer.

At the same time, we should point out the limitation of online consultation, such as the qualification and certification of registered doctors, quality control of the service, and lack of physical examination. Therefore, online consultation cannot replace in-person medical service. It is recommended that patients should go to the clinic for their first time medical encounter to prevent miss of important data. Internet medical service may be more advantageous for follow-up patients.

In less developed countries and regions, an imbalance of medical resources exists and will be further aggravated by the epidemic control policy under the current situation. In developed countries and regions, society shutdown has resulted in difficult access to medical services for patients. Some countries are returning to normal order from the epidemic, but they are still required to keep a certain social distance to prevent the spread of the virus, and thus they need an alternative way to provide medical services: the Internet-based medical model is a reliable choice (17).

Our Internet-based multidisciplinary management model has achieved promising results in patients with GU cancer in China in the context of the COVID-19 pandemic. We believe that our new model of cancer survivor management, the application, and the experience of Internet-based multidisciplinary management in patients with GU cancer can provide references for our peers all over the world during this COVID-19 pandemic.

We hope our overseas colleagues and medical industries work together and explore more convenient and effective Internet-based multidisciplinary management models to help more cancer survivors who are delayed in receiving healthcare due to the COVID-19 pandemic.

## Data availability statement

The original contributions presented in the study are included in the article/supplementary material, further inquiries can be directed to the corresponding authors.

## Ethics statement

The study was approved by the Research Ethics Committee of Shanghai Cancer Center, Fudan University, China according to the provisions of the Declaration of Helsinki (as revised in Fortaleza, Brazil, October 2013). The written informed consent was obtained from all individual participants included in this study, in accordance with the Declaration of Helsinki.

## Author contributions

YZ and SJ: project development and manuscript writing. HF, WY, and XQ: manuscript editing. HZ and XL: data collection. CG, WG, and FW: data analysis. DY: project development and manuscript editing. All authors contributed to the article and approved the submitted version.

## Funding

This study was funded by grants from the Shanghai Municipal Health Commission (Nos. 2018ZHYL0201 and 2019SY074), the Shanghai Science and Technology Committee (No. 18511108000), Shanghai Anti-Cancer Association Eyas Project (No. SACA-CY20A01), Shanghai Rising Star Program Sailing Project (Grant No. 22YF1408500), and Fudan University Shanghai Cancer Center Fund (No. YJQN202104).

## Conflict of interest

The authors declare that the research was conducted in the absence of any commercial or financial relationships that could be construed as a potential conflict of interest.

## Publisher's note

All claims expressed in this article are solely those of the authors and do not necessarily represent those of their affiliated organizations, or those of the publisher, the editors and the reviewers. Any product that may be evaluated in this article, or claim that may be made by its manufacturer, is not guaranteed or endorsed by the publisher.
